# Peer-Assisted History-Taking Groups: A Subjective Assessment of their Impact Upon Medical Students' Interview Skills

**DOI:** 10.3205/zma001112

**Published:** 2017-08-15

**Authors:** Katharina Eva Keifenheim, Ernst Richard Petzold, Florian Junne, Rebecca Sarah Erschens, Natalie Speiser, Anne Herrmann-Werner, Stephan Zipfel, Martin Teufel

**Affiliations:** 1 University Hospital of Tübingen, Department of Psychosomatic Medicine and Psychotherapy, Tübingen, Germany; 2 RWTH Aachen, Department of Psychosomatics and Psychotherapeutic Medicine, Aachen, Germany; 3 University of Duisburg-Essen, LVR-University Hospital Essen, Department of Psychosomatic Medicine and Psychotherapy, Essen, Germany

**Keywords:** Undergraduate medical education, medical students, physician-patient relationship, interpersonal relations, medical history taking, peer-assisted learning

## Abstract

**Background and Objectives:** Among the clinical skills needed by all physicians, history taking is one of the most important. The teaching model for peer-assisted history-taking groups investigated in the present study consists of small-group courses in which students practice conducting medical interviews with real patients. The purpose of this pilot study was to investigate the expectations, experiences, and subjective learning progress of participants in peer-assisted history-taking groups.

**Methods: **The 42 medical student participants completed a 4-month, peer-assisted, elective history-taking course, which both began and ended with a subjective assessment of their interview skills by way of a pseudonymized questionnaire. Measures comprised the students’ self-assessment of their interview skills, their expectations of, and their experiences with the course and especially with the peer tutors.

**Results: **Medical students’ most important motivations in attending peer-assisted history-taking groups were becoming able to complete a structured medical interview, to mitigate difficult interviewing situations, and to address patients’ emotional demands appropriately. By the end of the course, students’ self-assessment of both their interview skills and management of emotional issues improved significantly. Students especially benefitted from individual feedback regarding interview style and relationship formation, as well as generally accepted and had their expectations met by peer tutors.

**Conclusions: **To meet the important learning objectives of history-taking and management of emotional issues, as well as self-reflection and reflection of student–patient interactions, students in the field greatly appreciate practicing medical interviewing in small, peer-assisted groups with real patients. At the same time, peer tutors are experienced to be helpful and supportive and can help students to overcome inhibitions in making contact with patients.

## Zusammenfassung

**Hintergrund und Fragestellung: **Die Anamneseerhebung ist eine der wichtigsten klinischen Fertigkeiten jedes künftigen Arztes. Die folgende Studie befasst sich mit peer-tutorengestützten Anamnesegruppen. In diesem Format üben Medizinstudierende die Anamneseerhebung in Kleingruppen und mit realen Patienten. Ziel der Studie ist eine Untersuchung der Erwartungen, Erfahrungen und des subjektiven Kompetenzzuwachses der Teilnehmer an solchen peer-tutorenstützten Anamnesegruppen.

**Methodik: **42 Medizinstudierende, die über 4 Monate an einer peer-tutorengestützten Anamnesegruppe als Wahlfach teilnahmen, wurden zu Beginn und Ende des Kurses nach der subjektiven Einschätzung ihrer Anamnesefertigkeiten gefragt. Es wurde ein pseudonymisierter Fragebogen verwendet. Neben der subjektiven Kompetenz in der Erhebung einer Anamnese wurden auch Erwartungen an und Erfahrungen mit dem Kurs und speziell den Peer-Tutoren erfragt.

**Ergebnisse: **Hauptmotivation, weshalb Medizinstudierende sich für tutorengestützte Anamnesegruppen als Wahlfach entscheiden, ist der Wunsch, eine strukturierte Anamneseerhebung zu erlernen. Außerdem haben die Studierenden das Ziel, mit schwierigen Situationen im Interview umgehen zu lernen und die emotionalen Bedürfnisse von Patienten angemessen beantworten zu können. Am Ende des Kurses hatte sich die Selbsteinschätzung der Studierenden hinsichtlich ihrer subjektiven Anamnesefertigkeiten und ihres Umgang mit Gefühlen signifikant verbessert. Die Studierenden profitierten besonders von individuellem Feedback zu ihrem Interviewstil und Beziehungsaufbau. Die Peer-Tutoren wurden sehr gut akzeptiert und erfüllten die Erwartungen der Teilnehmer.

**Schlussfolgerung: **Studierende schätzen es sehr, die Anamneseerhebung in peer-gestützten Kleingruppen und mit realen Patienten zu üben. Dies kann sie beim Erreichen wichtiger Lernziele wie einer strukturierten Anamneseerhebung und dem Umgang mit emotionalen Situationen, aber auch in ihrer Selbstreflektion und Reflektion der Student-Patient-Beziehung, unterstützen. Die Peer-Tutoren wurden von den Teilnehmern als hilfreich und unterstützend erlebt und können Studierenden dabei helfen, Hemmungen beim Kontaktaufbau mit realen Patienten zu überwinden.

## Introduction

During the past 30 years, history taking and communication skills programmes have become cornerstones of medical education. Predominantly taught in small group courses that involve role-play and simulation [1], history taking programs in medical school focus on equipping students with measurable interview skills, especially in terms of completeness of content, structure, and questioning techniques. However, only a few approaches address aspects of relationship formation, involve real patients, or offer peer tutors [1], [2], [3], [4], [5], [6], though peer teaching is widely used in other fields of medical education [7] such as basic science courses, clinical skills training or problem-based learning courses. 

The model for practicing medical interviewing in peer-assisted history-taking groups investigated in the present study emerged from a student initiative to support patient-centered approaches in undergraduate medical education. Emerging in Germany in 1969, history-taking groups have since spread across Europe, and today, peer-assisted history-taking groups are offered at medical faculties in Germany, Austria, Switzerland, and Denmark. As in similar projects undertaken in eastern European countries, these group courses are likely to occur in the context of students’ self-organization and apart from the regular curriculum. Yet, they have increasingly been offered as elective courses embedded within a longitudinal communication skills curriculum [8], [9]. 

History-taking groups consist of about six to eight student participants and two peer tutors. Group sessions take place once per week, typically in the evening. In each session, one of the students takes the medical history of a real patient – an inpatient from a different hospital department each week – in front of the group of students. The interview is followed by a feedback session and group discussion without the patient. Feedback sessions focus on not only technical aspects of the interview, but also student-patient interaction and the student’s management of the patient’s emotional demands – for example, anxiety, grief, and responses to chronic disease [8]. Peer tutors involved in the process are trained in special workshops [10], [11] and are supervised regularly.

In history-taking groups, learning objectives include improving students’ interview skills, enabling them to foster confident relationships with patients, increasing their awareness of patient-centered approaches, facilitating empathy, and encouraging self-reflection [12], [13]. Additionally, students gain awareness of interpersonal dimensions and learn how to actively and authentically participate in conversations with patients. Beyond improving interview management, history-taking groups also support medical students’ development as professional physicians. During this process, students may acquire and refine fundamental professional values as well as ethical and social attitudes [12], [13], [14], [15], [16], [17], [18], [19], [20], [21], [22], [23]. 

Research has shown that providing students with the opportunity to work in small groups with peer tutors is not only highly appreciated, but can also improve students’ clinical skills [4], [7], [24]. Moreover, peer tutors can be powerful role models who support not only knowledge and clinical skills, but also attitudinal learning [7], [24], [25], [26], [27]. Several studies have suggested that participating in a peer-assisted learning project poses substantial benefits for students [4], [24], [28], [29], [30], [31]. Briefly, the effectiveness of peer tutors can be linked to their cognitive and social congruence with learners [7], [32]; research has shown that peer tutors have a better understanding of learners’ prior knowledge and of possible challenges and can recall personal approaches used to more easily master these challenges by way of what is termed *cognitive congruence* [7], [33]. Peer tutors furthermore create a comfortable, safe educational atmosphere and have been found to be particularly supportive and encouraging and to reduce learner anxiety, by way of what is called *social congruence* [7], [32], [33], [34]. To date, however, peer-assisted learning in history-taking groups has been evaluated in very few published reports [9], [12], [13], [20], [21], [35].

Peer tutors in history-taking groups facilitate feedback, moderate discussion, and manage group processes, which support students’ reflection upon themselves and upon student–patient relationship and interaction [19], [20], [21]. Peer tutors not only impart knowledge and skills regarding communicative competence and interview techniques, but moreover give structured feedback to participants. By realizing those functions, they also function as role models to participants and thereby influence attributes that students might emulate [14].

Yet, despite the widespread application of history-taking groups across Europe, few investigations have been conducted to clarify what attracts students to these groups and which skills or attitudes they develop therein. Due to this uncertainty, the longitudinal PGroWTH study (“Peer-Assisted, Group-Oriented Way of Teaching History-Taking”) was conducted to examine the following questions:

1. What do students expect from the teaching format of peer-assisted history-taking groups?
2. What do students expect from their peer tutors?
3. How do students describe their experiences with peer tutors?
4. How does students’ subjective assessment of their interview skills change throughout the course?

## Methods

### Study design

The 42 students who participated in the study completed an elective 4-month-long peer-assisted history-taking course held weekly in the evening for 2 h. Each group consisted of eight or nine participants and two peer tutors that were at least in their second year. If possible, tutors worked in mixed-gender tandems In the first session, peer tutors addressed basic interview skills and questioning techniques as well as the structure and content of medical interviews. In each of the following 13 sessions, one of the students interviewed a real patient in front of the group. By having a patient from varying departments (e.g., internal medicine, surgery, psychiatry, orthopedics, and gynecology) every week, interview models support a broad perspective. Afterwards, the student received feedback from both the group and the peer tutors on the structure and content of his or her interview, interview skills, if necessary his or her dealing with emotional issues and the formation of the physician–patient relationship. Feedback was followed by a group discussion moderated by the peer tutors, all of whom had received a didactic training as well as a specific training to qualify them to teach interview skills in preparation for the course. 

#### Instruments

We used a written questionnaire adapted from Hils (35) which is available at https://edoc.ub.uni-muenchen.de/10530/. Hils’ questionnaire consists of 75 (T0) and 91 (T1) items. Predominantly, multiple choice questions (MC) are used. She is also asking for suggestions and comments. 

In our adapted version, the following information were recorded at the beginning (T0; before session 1) and end (T1; after session 14) of the course:

- Students’ expectations of the course (T0);
- Students’ expectations of peer tutors (T0);
- Students’ experiences with their peer tutors (T1); and
- Students’ self-assessment of their current interview skills, subdivided into interviewing techniques and the management of emotional issues (T0; T1).

The questionnaires are published online and are available at Attachment 1 and Attachment 2. Questionnaires were pseudonymized and assignable by codes. In the section addressing expectations and experiences, students rated what they expected to learn from the course (T0), how important they found different possible functions of the tutors (T0), and to what extent tutors fulfilled those requirements (T1). To assess importance, a 7-point Likert scale was divided into three parts: *less important (1 and 2), important* (3–5), and *very important* (6 and 7). In the section addressing interview skills, students used another 7-point Likert scale to rate their ability to conduct certain skills. They were also invited to make free-text comments at the end of the questionnaire. 

#### Data analysis

Comparative data were analyzed using the Statistical Package for the Social Sciences version 21 (IBM, New York, NY, USA) and R 3.1.0 (Open Source; www.r-project.org). Items were clustered on scales when they exhibited sufficient internal consistency, which was reached when Cronbach’s alpha was >0.8. Scales at T0 and T1 were compared using the Wilcoxon rank-sum test for dependent samples, and descriptive data were analyzed using standard statistical methods. Since data were not normally distributed, the Bravais-Pearson correlation *r* was used to display effect sizes. According to Cohen [36], results should be interpreted as small (*r*>0.1), medium (*r*>0.3), and high (*r*>0.5). At the same time, a structured qualitative content analysis was conducted with the free-text comments using an inductive coding framework. 

## Results

### Sample 

The sample consisted of 42 students who participated in an elective peer-assisted history-taking course. The majority of the participants was female. For most of them, it was the first time to take part in a history-taking group. 76,1% of the participants were in the first two years (preclinical medical studies), only 23,9% were in the clinical stage. For a detailed description of the sample, see Table 1 (Tab. 1). 

#### Participants’ expectations of history-taking groups

Regarding students’ expectations of their participation in history-taking groups, see Table 2 (Tab. 2). 

More than 80% considered it “very important” to learn about structure and technique of the medical interview and to learn how to manage difficult and emotional situations, but only about 40% considered self-experience aspects “very important”.

In their free-text comments, students most frequently mentioned their wish for earlier contact with patients. 

#### What did participants expect from their student tutors, and which experiences did they gain?

**Figure 1:**
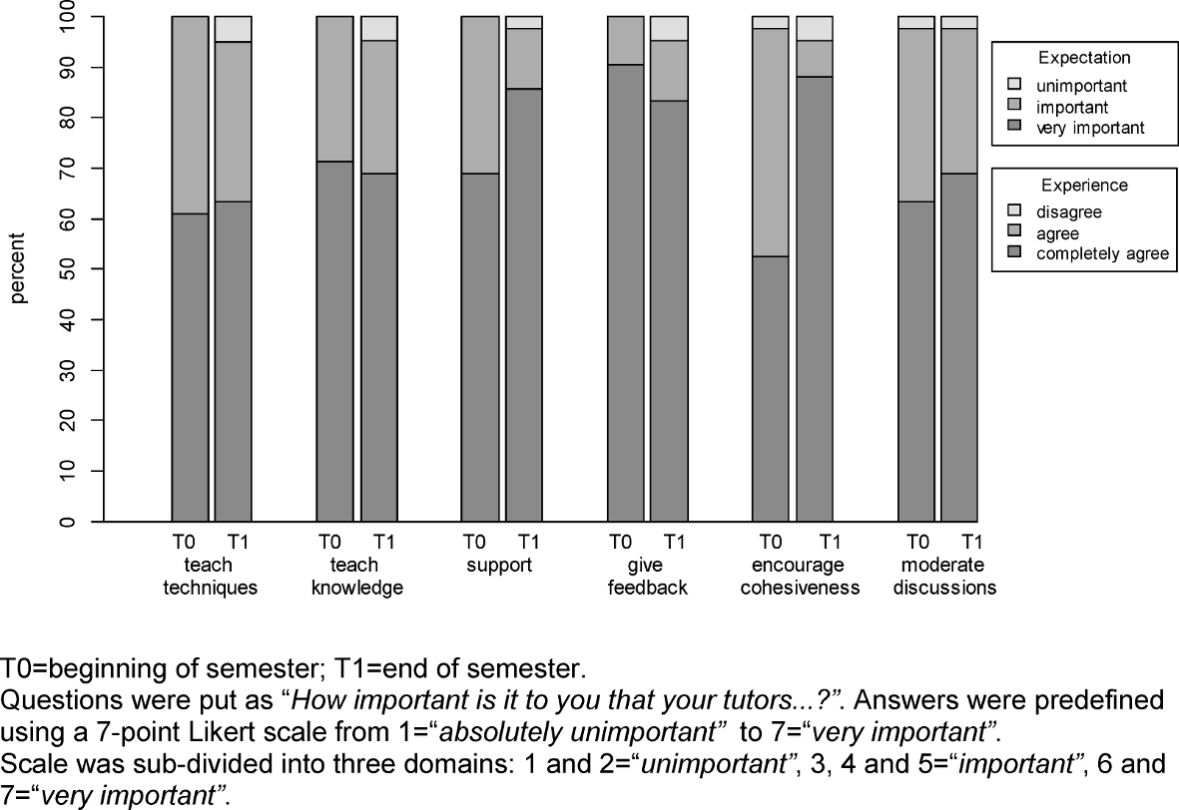
Participants’ expectations towards (T0) and experiences with (T1) their peer tutors

Participants rated how important (T0) they found the different possible functions of peer tutors suggested in the questionnaire and to what degree tutors fulfilled those tasks (T1). As results shown in Figure 1 (Fig. 1) indicate, participants most expected their tutors to provide them with feedback, which 90.48% deemed very important. Furthermore, 83.33% of participants completely agreed that tutors could accomplish that task very well. Surprisingly, only 60–70% of participants found it very important that tutors taught knowledge (71.43%) and techniques (60.98%), a task that 69.1% and 61.9% of participants completely agreed that tutors fulfilled very well. Although only 50–70% of participants deemed the tasks of supporting participants (69.05%) and fostering group cohesiveness (52.58%) to be very important, more than 85% of them completely agreed that the tutors performed those tasks (85.71% and 88.10%, respectively). 

#### Self-assessment of interview skills

**Figure 2:**
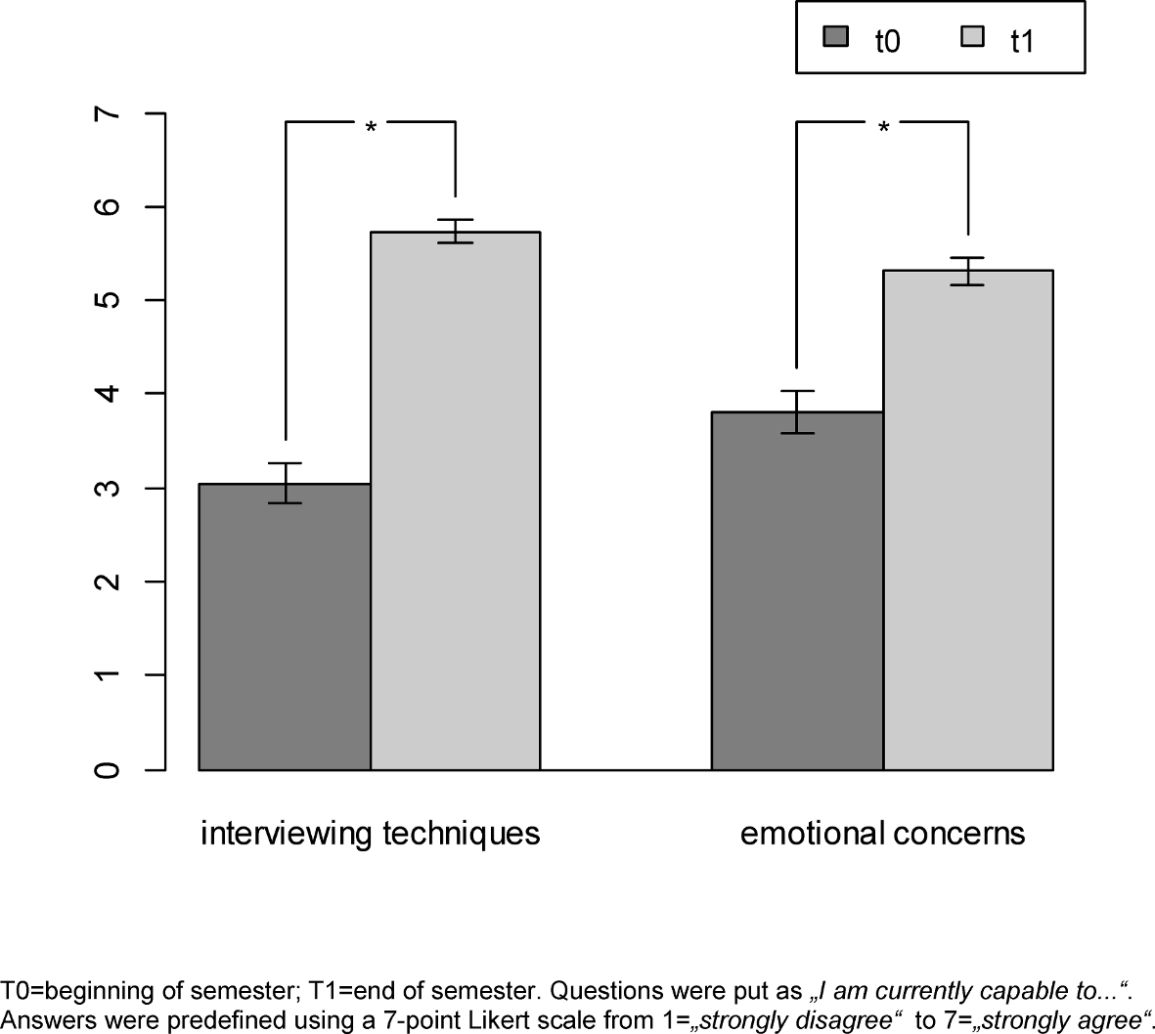
Participants’ self-assessment of interviewing skills

Items were clustered into interviewing skills (i.e., the structure and content of medical interviews, questioning style, and dealing with pauses) and the management of emotional issues (i.e., reflection upon personal emotions, dealing with patient’s emotional expressions, and keeping one’s distance). Students’ subjective interviewing skills improved significantly (*p*<.001; *Z*=-5.179; *r*=-0.799) from T0 (*M*_T0_=3.03) to T1 (*M*_T1_=5.73). As shown in Figure 2 (Fig. 2), a similar finding emerged regarding students’ management of emotional issues (*M*_T0_=3.72; *M*_T1_=5.37; *p*<.001; *Z*=-5.527; *r*=-0.853). 

Standardized effect sizes (Pearson’s correlation coefficient *r*) were high, which reflected high clinical effects apart from statistical significance.

#### Free-text comments

At the end of both questionnaires, we asked for suggestions and comments. Recurrent topics in free-text comments addressed real patients, learning situations, tutors’ functions, and balancing stress at university, as the following examples illustrate. About dealing with real patients, one participant reported, *“Participation helped me a lot to learn bedside manners and to lose my inhibitions with asking certain questions – for example, inquiring about the disease.”* Meanwhile, on the topic of learning situations, another participant said, *“It was possible to fully concentrate on the interview, without anxiety or pressure to perform.”* Concerning tutors’ functions, one student indicated that *“[t]utors gave extremely good feedback; they often noticed details that would have never attracted my attention. They didn’t only praise; they also gave quite critical feedback sometimes. But they were always supportive.”* Lastly, on the subject of balancing stress at university, one participant stated, *“The history-taking group was a pleasant and interesting addition to the usually highly theoretical studies. It was great fun for me, and I would recommend it to everyone.”*

Suggestions for improvement mostly included the wish for more theoretical input regarding medical history taking. 

## Discussion

To the best of our knowledge, PGroWTH was one of the first studies to investigate what students expect from elective history-taking groups, what they expect from peer tutors, how they describe their experiences with peer tutors, and which skills they subjectively acquire during the course.

Regarding what students expect from the teaching format of peer-assisted history-taking groups, our results confirm that students greatly desire early contact with patients and scenarios with clinical relevance. Students’ expectations clarify that their needs go beyond those fulfilled by lectures, instructions, and role-play and they highlight the necessity for similar learning situations close to reality. Other than to gain knowledge and learn new techniques, students also often look for help with difficult interpersonal situations, for advice on dealing with the expression of emotions, and especially for useful, individualized feedback. Students moreover seek opportunities for interacting with patients in authentic environments, namely their future workplaces, and therefore appreciate learning situations in the hospital and with real patients. These results are consistent with the literature: Participating in history-taking groups can also help students to better understand their role as future professionals when dealing with patients and when having discussions with colleagues; for instance, active participation and practice give students a feeling of self-efficacy. 

Concerning what students expect from peer tutors and how they describe their experiences with tutors, participants reported that they expected their tutors to impart knowledge and demonstrate techniques, yet moreover require their tutors’ personal feedback on their performance. Alongside recent literature [7], [14], [32], [34], our findings illustrate that tutors can fulfill these expectations to a great extent. In hindsight, participants found their tutors’ feedback to be motivating, and they appreciated that their tutors supported them individually and reinforced group cohesiveness. This perception of tutors supports recent findings postulating that tutors can be powerful role models who facilitate and support students’ socialization into their current roles and future professions [7], [14], [24], [25], [26], [32], [36]. 

Lastly, regarding how students’ subjective interview skills changed throughout the course, by evaluating students’ self-assessment at the beginning and end of the course, we found a significant increase in their perceived interview skills and management of emotional issues, including self-reflection. It should be emphasized that there were not only statistically significant effects, but that the resulting effect sizes can be considered quite large, thereby reflecting high clinical and practical relevance. As history-taking groups last for two semesters at many faculties in Germany, future research should investigate if the second semester achieves further improvement, as significant effects could already be shown after one semester in our study. 

Students in German-speaking countries and increasingly across Europe take part in history-taking groups in their free time and typically in addition to a demanding, time-consuming curriculum. Increasingly, history-taking groups are offered as elective courses and are part of longitudinal communication skills curricula. Clearly, peer-assisted history-taking groups provide students with something that they cannot access in regular curricula. Results from this study suggest that part of the attractiveness of these groups might be explained by their providing early contact with real patients and the opportunity to receive personal, useful feedback, to acquire strategies for managing difficult and/or emotional situations with patients, and to practice in a positive, comfortable, and safe learning environment. 

## Limitations

To our knowledge, our PGroWTH study is one of the first to investigate students’ expectations of, experiences with, and learning progress with peer-assisted history-taking groups. There are several limitations regarding our study, however. This pilot study followed an exploratory mixed-methods design to investigate the above dimensions, although future studies on the subject might benefit from larger sample sizes, objective measurements of students’ skills and self-assessment, and follow-up interviews within a controlled study design. Especially objective evaluation of interview skills will be essential [37]. In our study, confounding variables in the interim between the first and final sessions might have influenced students’ attitudes and their self-assessment of communication skills.

Since participants in the project formed a self-selected group, they may differ from their colleagues in terms of attitudes toward patient-centered approaches, small-group teaching, and student tutors. Although the application of a control group might seem reasonable, in our pilot study we decided against it. Since history-taking groups are optional, they attract a certain subgroup that cannot be compared to a control group of the standard cohort. At the same time, history-taking groups go beyond imparting mere skills and techniques. In that sense, the present pilot study does not examine the superiority of a certain teaching method, but investigates motivations in taking part in, expectations of, and experiences with peer-assisted history-taking groups. 

## Conclusion

This study showed that history-taking groups improve participants’ self-assessment of interview skills as well as fulfill their expectations. As elective peer-assisted courses, history-taking groups are part of longitudinal communication skills curricula that attract students early in undergraduate medical education. 

Medical school teachers and curriculum planners should consider both the early formation of attitudes and identification of role models in medical school education. It is advised that communication skills courses entail more than only skills; instead, such formats should also address aspects such as physician–patient relationships, patient-centeredness, and reflection upon physician–patient interaction. Early practical relevance, the early application of peer tutors, and an introduction to patient-centered approaches in preclinical years should therefore be goals in future curriculum development. 

## Acknowledgements

The authors would like to thank the peer tutors who were so enthusiastic and dedicated to the project. 

## Competing interests

The authors declare that they have no competing interests.

## Supplementary Material

Evaluation sheet medical history groups – member semester: T0 – in German

Evaluation sheet medical history groups – member semester: T1 – in German

## Figures and Tables

**Table 1:**
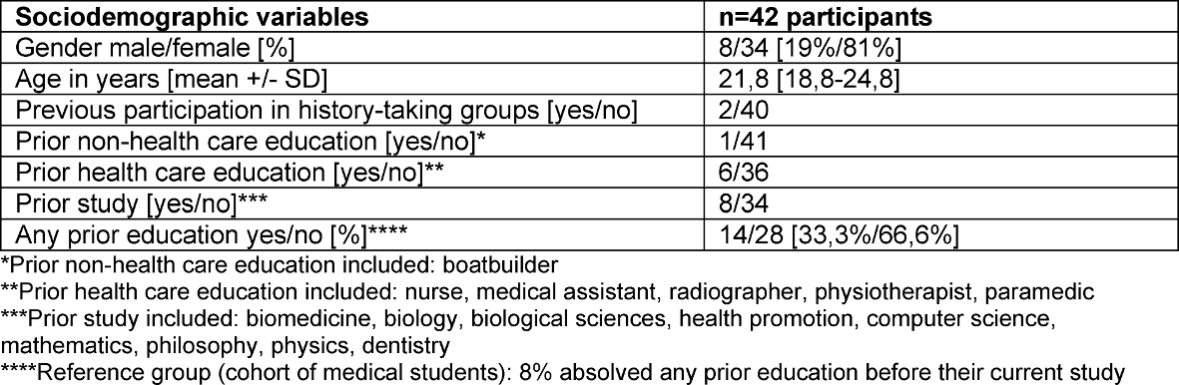
Sample description: Basic socio-demographic characteristics of participants

**Table 2:**
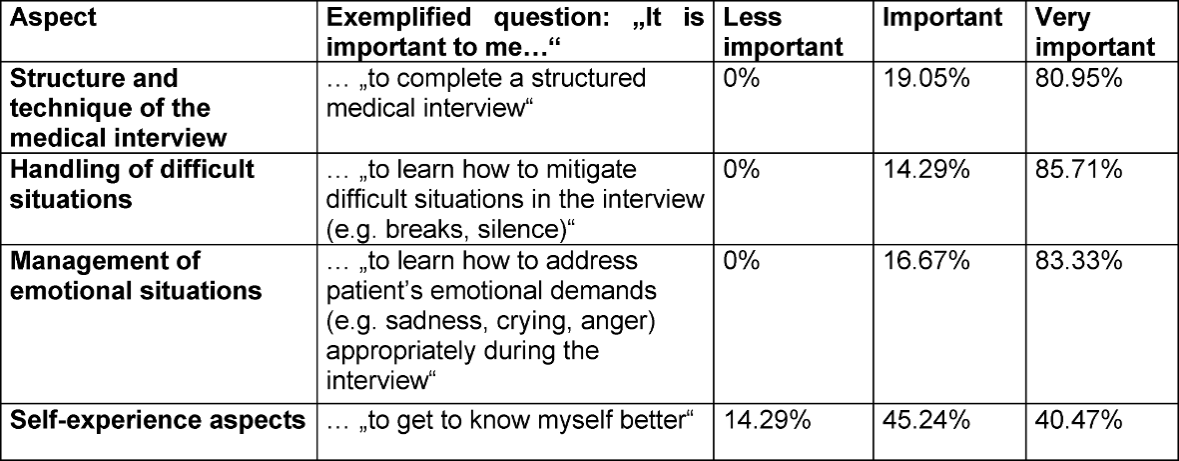
What do students expect from their participation in history-taking groups?
