# Supplementary material for: Peer-Assisted History-Taking Groups: A Subjective Assessment of their Impact Upon Medical Students' Interview Skills
Source: GMS J Med Educ. 2017 Aug 15;34(3):Doc35. doi: 10.3205/zma001112 (PMC5569984; doi:10.3205/zma001112)
Supplement: Evaluation sheet medical history groups – member semester: T0 – in German [file JME-34-35-s-001.pdf]

Evaluationsbogen Anamnesegruppen – Teilnehmer* Semester:___________ T0

Liebe Studierende,

gemeinsam mit Ihnen möchten wir die Ausbildung von angehenden Medizinern und Psychologen im Bereich Kommunikation und Interaktion verbessern. Deshalb bitten wir Sie herzlich, den folgenden Fragebogen auszufüllen. Am Ende des Semesters erhalten Sie einen zweiten Bogen von Ihren Tutoren. Die Fragebögen sind anonymisiert und Ihre Angaben können nicht Ihrer Person zugeordnet werden. Ebenso haben Ihre Angaben selbstverständlich keinen Einfluss auf Ihre Note.

Vielen Dank für Ihre Mitarbeit!

I. Code:

Teilnehmercode: Bitte generieren Sie sich einen Code. Diesen benötigen wir, um den Fragebogen, den Sie am Ende des Semesters ausfüllen werden, diesem Fragebogen zuzuordnen. Der Code besteht aus 5 Zeichen:

1. Anfangsbuchstabe des Vornamens Ihrer Mutter
2. Anfangsbuchstabe Ihres Vornamens
3. Anfangsbuchstabe der Stadt, in der Sie geboren sind
4. Ihr Geburtstag (nur der Tag, bei einstelligen Zahlen bitte eine 0 davorsetzen. Wenn Sie am 8. Mai geboren sind, schreiben Sie z.B. 08).

✐__________

Gruppencode: Welche Anamnesegruppe besuchen Sie im kommenden Semester? Bitte geben Sie von beiden Tutoren die Initialen des Vor- und Nachnamens an. Bei Anna Bauer und Julia Maier schreiben Sie z.B.: ABJM.

✐__________

II. Basisdaten

(2.1) Geschlecht: ☐ weiblich ☐ männlich

(2.2) Alter: ✐ _____ Jahre

(2.3) Studienfach: ☐ Medizin ☐ Psychologie

(2.4) Fachsemester ✐ _____ Semester

(2.5) Haben Sie zuvor schon einmal an einer Anamnesegruppe teilgenommen?

☐ ja ☐ nein

(2.6) Wenn ja, wie viele Semester haben Sie bisher an einer Anamnesegruppe teilgenommen? ✐___

(2.7) Haben Sie vorher eine andere Berufsausbildung/Studium begonnen oder abgeschlossen?

☐ ja, Studium ☐ ja , Berufausbildung

☐ nein

Wenn ja: Welche/Welches? ✐ ___________________

☐ abgeschlossen ☐ nicht abgeschlossen

* Im Folgenden ist immer nur die männliche Form gewählt; gemeint sind natürlich immer beide Geschlechter gleichermaßen.

Copyright © 2017, UKT, Innere Medizin VI/Abteilung Psychosomatische Medizin und Psychotherapie, Tübingen
Alle Rechte, insbesondere das Recht zur Vervielfältigung und Verbreitung sowie der Übersetzung vorbehalten. Kein Teil des Werkes darf in irgendeiner Form ohne schriftliche Genehmigung der Abteilung reproduziert oder unter Verwendung elektronischer Systeme gespeichert, verarbeitet, vervielfältigt oder verbreitet werden.

III. Selbsteinschätzung zur kommunikativen Kompetenz

Bitte kreuzen Sie das am ehesten Zutreffende an!

1	2	3	4	5	6	7
trifft nicht zu						trifft zu

Ich bin zum jetzigen Zeitpunkt in der Lage…	
(3.1) eine biopsychosoziale Anamnese unter Berücksichtigung der Sozial- und Familienanamnese strukturiert durchzuführen	① ② ③ ④ ⑤ ⑥ ⑦
(3.2) Überleitungen zwischen einzelnen Anamneseblöcken zu gestalten	① ② ③ ④ ⑤ ⑥ ⑦
(3.3) in Gesprächspausen ruhig zu bleiben und nach einiger Zeit ggf. neue Anknüpfungspunkte zu finden	① ② ③ ④ ⑤ ⑥ ⑦
(3.4) mit den verbalen und nonverbalen Gefühlsäußerungen des Patienten angemessen umzugehen	① ② ③ ④ ⑤ ⑥ ⑦
(3.5) meine eigenen Gefühle während des Gesprächs wahrzunehmen und angemessen damit umzugehen	① ② ③ ④ ⑤ ⑥ ⑦
(3.6) mich auf einen Patienten einzulassen, unter Wahrung der nötigen professionellen Distanz	① ② ③ ④ ⑤ ⑥ ⑦

IV. Erwartungen an die Anamnesegruppe

Bitte kreuzen Sie das am ehesten Zutreffende an!

1	2	3	4	5	6	7
trifft nicht zu						trifft zu

Es ist mir wichtig, durch die Teilnahme an einer Anamnesegruppe…	
(4.1) eine strukturierte Anamneseerhebung zu erlernen	① ② ③ ④ ⑤ ⑥ ⑦
(4.2) Techniken zur Gesprächsführung (z.B. Fragetechniken) zu erlernen	① ② ③ ④ ⑤ ⑥ ⑦
(4.3) zu lernen, wie man mit schwierigen Situationen (Schweigen, Pausen) im Anamnesegespräch umgehen kann	① ② ③ ④ ⑤ ⑥ ⑦
(4.4) zu lernen, mit den Emotionen des Patienten (z.B. Traurigkeit, Weinen, Ärger) im Anamnesegespräch angemessen umzugehen	① ② ③ ④ ⑤ ⑥ ⑦
(4.5) zu lernen, meine eigenen Gefühle im Umgang mit Patienten zu reflektieren	① ② ③ ④ ⑤ ⑥ ⑦
(4.6) einen professionellen Umgang mit dem Patienten zu erlernen	① ② ③ ④ ⑤ ⑥ ⑦
(4.7) mich selbst besser kennen zu lernen	① ② ③ ④ ⑤ ⑥ ⑦
(4.8) etwas über Zusammenhänge von Krankheit und Lebensgeschichte der Patienten zu lernen	① ② ③ ④ ⑤ ⑥ ⑦

Copyright © 2017, UKT, Innere Medizin VI/Abteilung Psychosomatische Medizin und Psychotherapie, Tübingen
Alle Rechte, insbesondere das Recht zur Vervielfältigung und Verbreitung sowie der Übersetzung vorbehalten. Kein Teil des Werkes darf in irgendeiner Form ohne schriftliche Genehmigung der Abteilung reproduziert oder unter Verwendung elektronischer Systeme gespeichert, verarbeitet, vervielfältigt oder verbreitet werden.

V. Erwartungen an die Tutoren

Bitte kreuzen Sie das am ehesten Zutreffende an!

1	2	3	4	5	6	7
trifft nicht zu						trifft zu

Es ist mir wichtig, dass die Tutoren…	
(5.1) mir Techniken zur Gesprächsführung (z.B. Fragetechniken, aktives Zuhören) nahe bringen	① ② ③ ④ ⑤ ⑥ ⑦
(5.2) Wissen (z.B. über Anamneseführung oder psychosomatische Zusammenhänge) vermitteln	① ② ③ ④ ⑤ ⑥ ⑦
(5.3) mich ermutigen und unterstützen	① ② ③ ④ ⑤ ⑥ ⑦
(5.4) mir Rückmeldung/Feedback geben	① ② ③ ④ ⑤ ⑥ ⑦
(5.5) die Gruppe „zusammenhalten“	① ② ③ ④ ⑤ ⑥ ⑦
(5.6) die Gruppendiskussion moderieren	① ② ③ ④ ⑤ ⑥ ⑦

VI. Einstellungen

Bitte kreuzen Sie das am ehesten Zutreffende an!

1	2	3	4	5	6	7
trifft nicht zu						trifft zu

Ich finde es im Allgemeinen wichtig, …	
(6.1) neben der Symptomatik auch etwas über die Lebensgeschichte und die aktuelle Lebenssituation des Patienten zu erfahren	① ② ③ ④ ⑤ ⑥ ⑦
(6.2) auf Zusammenhänge zwischen der Symptomatik/Krankengeschichte und der Lebensgeschichte des Patienten zu achten	① ② ③ ④ ⑤ ⑥ ⑦
(6.3) im Gespräch darauf zu achten, was der Patient nonverbal und indirekt mitteilt	① ② ③ ④ ⑤ ⑥ ⑦
(6.4) zu erfahren, worin der Patient selbst die Ursache seiner Erkrankung sieht	① ② ③ ④ ⑤ ⑥ ⑦
(6.5) eigene Eindrücke, Gefühle und Fantasien aus dem Patientengespräch wahrzunehmen und zu reflektieren	① ② ③ ④ ⑤ ⑥ ⑦

Copyright © 2017, UKT, Innere Medizin VI/Abteilung Psychosomatische Medizin und Psychotherapie, Tübingen
Alle Rechte, insbesondere das Recht zur Vervielfältigung und Verbreitung sowie der Übersetzung vorbehalten. Kein Teil des Werkes darf in irgendeiner Form ohne schriftliche Genehmigung der Abteilung reproduziert oder unter Verwendung elektronischer Systeme gespeichert, verarbeitet, vervielfältigt oder verbreitet werden.

VII. Kommentare und Anregungen
(Anregungen auch über diesen Fragebogen sind willkommen)

Copyright © 2017, UKT, Innere Medizin VI/Abteilung Psychosomatische Medizin und Psychotherapie, Tübingen
Alle Rechte, insbesondere das Recht zur Vervielfältigung und Verbreitung sowie der Übersetzung vorbehalten. Kein Teil des Werkes darf in irgendeiner Form ohne schriftliche Genehmigung der Abteilung reproduziert oder unter Verwendung elektronischer Systeme gespeichert, verarbeitet, vervielfältigt oder verbreitet werden.
