# Supplementary material for: Peer-Assisted History-Taking Groups: A Subjective Assessment of their Impact Upon Medical Students' Interview Skills
Source: GMS J Med Educ. 2017 Aug 15;34(3):Doc35. doi: 10.3205/zma001112 (PMC5569984; doi:10.3205/zma001112)
Supplement: Evaluation sheet medical history groups – member semester: T1 – in German [file JME-34-35-s-002.pdf]

Evaluationsbogen Anamnesegruppen – Teilnehmer* Semester:__________ T1

Liebe Studierende,

wie zu Beginn des Semesters erhalten Sie heute erneut einen Fragebogen zu den Anamnesegruppen. Bitte füllen Sie diesen aus. Ihre Rückmeldungen helfen uns, die Ausbildung von angehenden Medizinern und Psychologen im Bereich Kommunikation und Interaktion weiter zu verbessern.

Vielen Dank für ihre Mitarbeit!

I. Code:

Zu Beginn des Semesters haben Sie sich einen Code generiert. Diesen benötigen wir, um den Fragebogen, den Sie jetzt ausfüllen werden, diesem ersten Fragebogen zuzuordnen. Zur Erinnerung: Der Code besteht aus 5 Zeichen:

1. Anfangsbuchstabe des Vornamens Ihrer Mutter
2. Anfangsbuchstabe Ihres Vornamens
3. Anfangsbuchstabe der Stadt, in der Sie geboren sind
4. Ihr Geburtstag (nur der Tag, bei einstelligen Zahlen bitte eine 0 davorsetzen. Wenn Sie am 8. Mai geboren sind, schreiben Sie z.B. 08).

✐__________

Gruppencode: Welche Anamnesegruppe haben Sie besucht? Bitte geben Sie von beiden Tutoren die Initialen des Vor- und Nachnamens an. Bei Anna Bauer und Julia Maier schreiben Sie z.B.: ABJM.

✐__________

II. Selbsteinschätzung zur kommunikativen Kompetenz

Bitte kreuzen Sie das am ehesten Zutreffende an!

1	2	3	4	5	6	7
trifft nicht zu						trifft zu

Ich bin zum jetzigen Zeitpunkt in der Lage…	
(2.1) eine biopsychosoziale Anamnese unter Berücksichtigung der Sozial- und Familienanamnese strukturiert durchzuführen	① ② ③ ④ ⑤ ⑥ ⑦
(2.2) den Patienten angemessen zu begrüßen, mich vorzustellen und die Anamnese zu beginnen	① ② ③ ④ ⑤ ⑥ ⑦
(2.3) Überleitungen zwischen einzelnen Anamneseblöcken zu gestalten	① ② ③ ④ ⑤ ⑥ ⑦
(2.4) Gesprächspausen auszuhalten und nach einiger Zeit ggf. neue Anknüpfungspunkte zu finden	① ② ③ ④ ⑤ ⑥ ⑦
(2.5) das Anamnesegespräch aktiv zu beenden und mich zu verabschieden	① ② ③ ④ ⑤ ⑥ ⑦
(2.6) ein Anamnesegespräch mit problematischen Patienten zu führen	① ② ③ ④ ⑤ ⑥ ⑦
(2.7) mit den verbalen und nonverbalen Gefühlsäußerungen des Patienten angemessen umzugehen	① ② ③ ④ ⑤ ⑥ ⑦
(2.8) meine eigenen Gefühle während des Gesprächs wahrzunehmen und angemessen damit umzugehen	① ② ③ ④ ⑤ ⑥ ⑦
(2.9) mich auf einen Patienten einzulassen, unter Wahrung der nötigen professionellen Distanz	① ② ③ ④ ⑤ ⑥ ⑦

* Im Folgenden ist immer nur die männliche Form gewählt; gemeint sind natürlich immer beide Geschlechter gleichermaßen.

Copyright © 2017, UKT, Innere Medizin VI/Abteilung Psychosomatische Medizin und Psychotherapie, Tübingen
Alle Rechte, insbesondere das Recht zur Vervielfältigung und Verbreitung sowie der Übersetzung vorbehalten. Kein Teil des Werkes darf in irgendeiner Form ohne schriftliche Genehmigung der Abteilung reproduziert oder unter Verwendung elektronischer Systeme gespeichert, verarbeitet, vervielfältigt oder verbreitet werden.

III. a) Lernfortschritt in der Anamnesegruppe

Bitte kreuzen Sie das am ehesten Zutreffende an!

1	2	3	4	5	6	7
trifft nicht zu						trifft zu

**Ich habe durch die Teilnahme an der Anamnesegruppe…**	
(3.1) gelernt, eine strukturierte Anamnese zu erheben	① ② ③ ④ ⑤ ⑥ ⑦
(3.2) Techniken zur Gesprächsführung erlernt	① ② ③ ④ ⑤ ⑥ ⑦
(3.3) etwas über Krankheitsbilder gelernt	① ② ③ ④ ⑤ ⑥ ⑦
(3.4) gelernt, wie ich mit schwierigen Situationen im Anamnesegespräch umgehen kann	① ② ③ ④ ⑤ ⑥ ⑦
(3.5) gelernt, mit den Emotionen des Patienten im Anamnesegespräch angemessen umzugehen	① ② ③ ④ ⑤ ⑥ ⑦
(3.6) gelernt, meine eigenen Gefühle im Umgang mit Patienten besser wahrzunehmen und zu reflektieren	① ② ③ ④ ⑤ ⑥ ⑦
(3.7) mich selbst besser kennen gelernt	① ② ③ ④ ⑤ ⑥ ⑦
(3.8) Ängste und Hemmungen gegenüber Patienten abbauen können	① ② ③ ④ ⑤ ⑥ ⑦
(3.9) etwas über die Zusammenhänge von Kranken- und Lebensgeschichte der Patienten gelernt	① ② ③ ④ ⑤ ⑥ ⑦
(3.10) gelernt, mich in andere Menschen besser einzufühlen	① ② ③ ④ ⑤ ⑥ ⑦
(3.11) gelernt, mich auf einen Patienten einzulassen und dabei eine professionelle Distanz zu wahren	① ② ③ ④ ⑤ ⑥ ⑦

Copyright © 2017, UKT, Innere Medizin VI/Abteilung Psychosomatische Medizin und Psychotherapie, Tübingen
Alle Rechte, insbesondere das Recht zur Vervielfältigung und Verbreitung sowie der Übersetzung vorbehalten. Kein Teil des Werkes darf in irgendeiner Form ohne schriftliche Genehmigung der Abteilung reproduziert oder unter Verwendung elektronischer Systeme gespeichert, verarbeitet, vervielfältigt oder verbreitet werden.

III. b) Lernerfahrungen in der Anamnesegruppe

Bitte kreuzen Sie das am ehesten Zutreffende an!

1	2	3	4	5	6	7
trifft nicht zu						trifft zu

Ich habe in der Anamnesegruppe besonders profitiert…	
(3.12) von der Anamnese, die ich selbst geführt habe	① ② ③ ④ ⑤ ⑥ ⑦
(3.13) von den Rückmeldungen der Teilnehmer zu der von mir geführten Anamnese	① ② ③ ④ ⑤ ⑥ ⑦
(3.14) von den Rückmeldungen der Tutoren zu der von mir geführten Anamnese	① ② ③ ④ ⑤ ⑥ ⑦
(3.15) von der Gruppendiskussion zu der von mir geführten Anamnese	① ② ③ ④ ⑤ ⑥ ⑦
(3.16) vom Beobachten der Anamnesegespräche der anderen Teilnehmer	① ② ③ ④ ⑤ ⑥ ⑦
(3.17) von den Rückmeldungen der Teilnehmer zu den Anamnesegesprächen insgesamt	① ② ③ ④ ⑤ ⑥ ⑦
(3.18) von den Rückmeldungen der Tutoren zu den Anamnesegesprächen insgesamt	① ② ③ ④ ⑤ ⑥ ⑦
(3.19) von den Gruppendiskussionen zu den Anamnesegesprächen insgesamt	① ② ③ ④ ⑤ ⑥ ⑦
(3.20) von der Vermittlung von Wissen und Techniken zur Gesprächsführung durch die Tutoren	① ② ③ ④ ⑤ ⑥ ⑦
(3.21) vom Input der Tutoren insgesamt	① ② ③ ④ ⑤ ⑥ ⑦

IV. Erfahrungen mit den Tutoren

Bitte kreuzen Sie das am ehesten Zutreffende an!

1	2	3	4	5	6	7
trifft nicht zu						trifft zu

Die Tutoren haben…	
(4.1) mir beigebracht, eine vollständige und strukturierte biopsychosoziale Anamnese zu erheben	① ② ③ ④ ⑤ ⑥ ⑦
(4.2) mir Techniken zur Gesprächsführung vermittelt	① ② ③ ④ ⑤ ⑥ ⑦
(4.3) mir Anregungen und Tipps gegeben	① ② ③ ④ ⑤ ⑥ ⑦
(4.4) Wissen und praktische Fertigkeiten vermittelt	① ② ③ ④ ⑤ ⑥ ⑦
(4.5) mich ermutigt und unterstützt	① ② ③ ④ ⑤ ⑥ ⑦
(4.6) einen Raum geschaffen, in dem Kritik möglich war	① ② ③ ④ ⑤ ⑥ ⑦
(4.7) die Gruppe zusammengehalten	① ② ③ ④ ⑤ ⑥ ⑦
(4.8) die Gruppendiskussion moderiert	① ② ③ ④ ⑤ ⑥ ⑦
(4.9) Gruppenprozesse angesprochen und erklärt	① ② ③ ④ ⑤ ⑥ ⑦
(4.10) bei Auseinandersetzungen in der Gruppe einzelne Teilnehmer geschützt	① ② ③ ④ ⑤ ⑥ ⑦

Copyright © 2017, UKT, Innere Medizin VI/Abteilung Psychosomatische Medizin und Psychotherapie, Tübingen
Alle Rechte, insbesondere das Recht zur Vervielfältigung und Verbreitung sowie der Übersetzung vorbehalten. Kein Teil des Werkes darf in irgendeiner Form ohne schriftliche Genehmigung der Abteilung reproduziert oder unter Verwendung elektronischer Systeme gespeichert, verarbeitet, vervielfältigt oder verbreitet werden.

(4.11) mir geholfen, mein Verhalten gegenüber Patienten besser zu verstehen	① ② ③ ④ ⑤ ⑥ ⑦

V. Einstellungen

Bitte kreuzen Sie das am ehesten Zutreffende an!

**1**	**2**	**3**	**4**	**5**	**6**	**7**
trifft nicht zu						trifft zu

**Ich finde es im Allgemeinen wichtig, …**	
(5.1) neben der Symptomatik auch etwas über die Lebensgeschichte und die aktuelle Lebenssituation des Patienten zu erfahren	① ② ③ ④ ⑤ ⑥ ⑦
(5.2) auf Zusammenhänge zwischen der Symptomatik/Krankengeschichte und der Lebensgeschichte des Patienten zu achten	① ② ③ ④ ⑤ ⑥ ⑦
(5.3) im Gespräch darauf zu achten, was der Patient nonverbal und indirekt mitteilt	① ② ③ ④ ⑤ ⑥ ⑦
(5.4) zu erfahren, worin der Patient selbst die Ursache seiner Erkrankung sieht	① ② ③ ④ ⑤ ⑥ ⑦
(5.5) eigene Eindrücke, Gefühle und Fantasien aus dem Patientengespräch wahrzunehmen und zu reflektieren	① ② ③ ④ ⑤ ⑥ ⑦
(5.6) dass die Gesprächsführung im Studium vermittelt wird und geübt werden kann	① ② ③ ④ ⑤ ⑥ ⑦

VI. Kommentare und Anregungen

(Anregungen auch über diesen Fragebogen sind willkommen)

Copyright © 2017, UKT, Innere Medizin VI/Abteilung Psychosomatische Medizin und Psychotherapie, Tübingen
Alle Rechte, insbesondere das Recht zur Vervielfältigung und Verbreitung sowie der Übersetzung vorbehalten. Kein Teil des Werkes darf in irgendeiner Form ohne schriftliche Genehmigung der Abteilung reproduziert oder unter Verwendung elektronischer Systeme gespeichert, verarbeitet, vervielfältigt oder verbreitet werden.

Copyright © 2017, UKT, Innere Medizin VI/Abteilung Psychosomatische Medizin und Psychotherapie, Tübingen
Alle Rechte, insbesondere das Recht zur Vervielfältigung und Verbreitung sowie der Übersetzung vorbehalten. Kein Teil des Werkes darf in irgendeiner Form ohne schriftliche Genehmigung der Abteilung reproduziert oder unter Verwendung elektronischer Systeme gespeichert, verarbeitet, vervielfältigt oder verbreitet werden.
